# Evaluation of the efficacy of nasal application of silver sulfadiazine for decolonization of patients with methicillin-resistant Staphylococcus aureus in hospitals

**DOI:** 10.1186/2047-2994-4-S1-O7

**Published:** 2015-06-16

**Authors:** LR Ferreira, MG Menegueti, GG Gaspar, MFI Silva, G David, NM Anselmo, MJF Gabriel, LM Sakamoto, R Martinez, F Bellissimo-Rodrigues

**Affiliations:** 1 HCFMRP/USP, Ribeirao Preto, Brazil; 2 FMRP/USP, Ribeirao Preto, Brazil

## Introduction

Previous nasal colonization is an important risk factor in the pathogenesis of infections caused by MRSA. Moreover, the formulation of mupirocin available in Brazil is inadequate for mucous membranes application, often causing intolerable side effects.

## Objectives

Evaluate the efficacy of intranasal application of silver sulfadiazine for decolonization of hospitalized patients carrying MRSA.

## Methods

This is a double-blind randomised study conducted at a tertiary-care university hospital, with approximately 700 beds. Adult in-patients from clinical and surgical wards were potentially eligible to the study if they had any Methicillin-Resistant Staphylococcus aureus isolated from any clinical specimen on the microbiology laboratory of the study facility. Exclusion criteria consisted of pregnant women, age <18 years, osteomyelitis, infections with implants and cystic fibrosis. Nasal colonization by MRSA was confirmed by means of a nasal swab selective culture. Patients were randomised by a pharmacist not involved on the data collection, and submitted to application intranasal of silver sulfadiazine 1% or placebo twice a day plus bath with clohexidine-degermant daily for five consecutive days. Nasal swab was repeated next day after end of decolonization. The rate of nasal MRSA decolonization were compared using the Fisher exact test.

## Results

Twenty-five patients were included. The median time from the day of hospitalization and inclusion in the study was 27 days (ranging from 7 to 85 days). The patient´s age median was 55.9 years old (ranging from 19 to 89 years). 16 male and 9 female patients. From the 25 patients initially included, 20 completed the full course of the study medication and had their cultures available after decolonization. Among the 20 patients, 5/12 (41.7%) patients in the silver sulfadiazine and 3/8 (37.5%) of the placebo group had negative nasal swab after decolonization (p = 0.74).

## Conclusion

According to these preliminary findings, silver sulfadiazine was not successful for decolonizing hospitalized patients carrying MRSA.

## Disclosure of interest

None declared.

